# Clinical Situations in Which the Diagnosis of Autism is Debatable: An Analysis and Recommendations

**DOI:** 10.1177/07067437211041469

**Published:** 2021-09-06

**Authors:** Pierre Defresne, Laurent Mottron

**Affiliations:** 1 Center for Autism Spectrum Disorders, SUSA Foundation, 54521 University of Mons, Belgium; 2 Faculty of Medicine, Psychiatry and Addictology Department, University of Montreal, Montreal, Quebec, Canada; 3 439501 CIUSSS-Nord-de-l’Ile de Montréal, 12368 Hospital Riviere-des-Prairies, Montreal, Quebec, Canada

**Keywords:** diagnosis, autism spectrum disorders, comorbidity, developmental transformations, nosology

## Abstract

The “autism spectrum disorder” (ASD) construct and its current diagnostic criteria have led to the inclusion of increasingly heterogeneous and decreasingly atypical individuals under its definition. This broad category, based on the polymorphic clinical expression of common genetic variants underpinning the risk of autism, is likely beneficial for certain individuals. However, determining the boundaries between ASD and typical individuals, as well as those with other neurodevelopmental conditions, remains an issue of which the importance is growing with the increase in ASD prevalence. We identified four clinical contexts associated with a questionable, poorly justified, or unhelpful ASD diagnosis: (1) those in which diagnostic instruments raise uncertainties, (2) in the context of a subclinical presentation, (3) when early autistic signs tend to fade away during development, and (4) when comorbidities are prominent. We argue that in certain cases, a diagnosis of ASD may not be the most suitable, timely, or helpful medical act and provide recommendations for clinical practice when facing such situations.

## Abrégé

Les critères actuels du « trouble du spectre autistique » (TSA) permettent d'inclure sous ce terme des individus de plus en plus nombreux, de plus en plus hétérogènes, et de moins en moins atypiques. Cette large catégorie pourrait se justifier par le polymorphisme de l'expression des variants génétiques communs sous-tendant le risque d'autisme. Elle peut aussi être bénéfique pour certains individus. Cependant, notre difficulté à déterminer la frontière entre les TSA et les individus typiques, ainsi qu'entre le TSA et d'autres conditions neuro-développementales, croit avec l'augmentation de la prévalence rapportée des TSA et pose des problèmes pratiques aux cliniciens. Nous avons identifié quatre contextes cliniques où la pose d'un diagnostic de TSA serait discutable, mal justifiée ou inutile : (1) quand les instruments diagnostiques standardisés donnent un résultat proche du seuil (2) dans les présentations infra-cliniques, (3) lorsque les signes autistiques initiaux s'estompent au cours du développement, et (4) lorsque les comorbidités sont cliniquement prédominantes. Nous présentons ici des recommandations pour la pratique clinique face à de telles situations où un diagnostic de TSA n'est ni l'acte médical ni plus approprié, ni le plus utile.

Recent diagnostic manuals have opted for a dimensional, “spectrum” approach to autism, assuming a continuum between subtle or ambiguous and more evident presentations. Nevertheless, no biological, brain imaging, neuropsychological, or genetic markers can currently unify the various presentations of autism as currently defined: the diagnosis of autism is still based on behavior. The recent introduction of clinical specifiers of intelligence, language, and adaptation in the definition of autism spectrum disorder (ASD) also contributed to an increased heterogeneity. However, despite standardized instruments to help diagnose autism, clinicians experience difficulties in establishing precise boundaries between autism, typical individuals, and other psychiatric/neurodevelopmental conditions.

The construct of the autism “spectrum” has been associated with clinical advantages. It precludes clinicians from classifying people into narrow, unreliable categories and has given disabled individuals the opportunity to access services and financial support that more restrictive diagnoses would not have allowed.

The prevalence of autistic individuals comparable to Kanner's seminal description has varied less over time than “administratively defined” autism cases^1^ and those that constitute the core of the spectrum.^2^ People with complex, atypical, or borderline conditions represent the majority of individuals composing the current reported prevalence. The increase in prevalence and the heterogeneity of needs represent a continuing challenge for organizing, allocating, and financing services.

We examine four clinical situations in which the current ASD diagnosis is problematical: (1) the limits of diagnostic criteria and instruments, (2) subclinical presentations, (3) developmental transformations, and (4) differential diagnosis.

## Diagnostic Criteria and Instruments

The diagnostic and statistical manual of mental disorders, 5th edition (DSM-5), ASD diagnosis now relies on the association of multiple, variable, disabling, and persisting deficits in social abilities and positive non-social signs. The association of elements of history, observation, and expertise is required, rather than the “checklist” application of DSM criteria. However, even applied in this way, it is unlikely that such “appropriate” use of the DSM criteria will fully resolve its role in creating heterogeneity. The steady increase in the prevalence and heterogeneity observed in recent decades suggests that the DSM recommendations, which aim to prevent the watering down of the condition, do not work.

“Diagnostic” instruments gather anamnestic and observational information on quantitative scales, with a cut-off score that determines the threshold at which the diagnosis is considered to have sufficient inter-rater reliability. They were never intended to be used without clinical judgment. However, although the psychometric properties of these instruments are considered to be good, their use raises certain issues: (1) clinical signs are shared with other neurodevelopmental conditions: reduced social relationships may be associated with most psychopathological conditions, insistence on sameness and rituals are common in anxious people, motor stereotypies may be confused with tics in Tourette syndrome,^3^ and sensory hypersensitivity is omnipresent in neurodevelopmental conditions, particularly Tourette syndrome and attention deficit hyperactivity disorder (ADHD)^4^; (2) severe specifier values may result in a positive diagnostic decision because most signs are expressed in a quantitative mode; (3) in the absence of biomarkers, we cannot distinguish false positives from deeply related conditions; (4) polythetic criteria result in a high number of clinically dissimilar combinations; (5) the interpretation of instrument scores close to the threshold may be challenging; and (6) diagnostic instruments do not propose a differential diagnosis and are uninformative for negative diagnoses.

Although the inter-rater reliability of experienced clinicians is excellent and possibly superior to that of instruments, a combination of the two approaches is generally recommended. The reliability of experienced clinicians is improved by their awareness of behavioral features that are underestimated using quantitative scales and symptoms checklists, and qualitatively more specific. The error margin of expertise may be more tolerable than the overinclusivity of standardized instruments, and their combination may result in increased specificity. However, as the ASD construct continues to expand, expertise itself may drift, and a new generation of experts may arise, mistaking autistic traits for the clinical presentation of autism.

## Subclinical Phenotype

Socio-communicative behaviors identified as “autistic traits” are continuously distributed across the population. The recognition of isolated or subtle autistic features in the relatives of autistic individuals has led to the consideration of a “broader autism phenotype,” encompassing features quantitatively or qualitatively insufficient to reach clinical recognition. The “Autism-Spectrum Quotient” and “Social Responsiveness Scale” are widely used to quantify such traits.

Whether or not subclinical individuals should be considered to belong to the same condition as clinically distinguishable individuals is, as yet, undecided. The indicators of a genetic predisposition may not straightforwardly correlate with the condition to which it predisposes.^5^ For example, in schizophrenia, preclinical manifestations and signs that display familial aggregation are separated from clinical schizophrenia by cognitive, behavioral, and neuroimaging differences.^6^

The diagnostic criteria for autism require significant impairment, as for most mental disorders. In autism, this requirement may confuse impairment and visibility. A certain level of visibility, present or past, should, however, be required for a categorical diagnosis.^7^ A related diagnostic issue is represented by situations in which individuals “camouflage” their difficulties in such a way that what we see is not what they experience. This possibility, although credible, has led to an explosion in requests for diagnostic services for individuals at the least evident end of the spectrum.

The inclination to include milder and earlier cases is a widespread practice in the field of medicine.^8^ Such definitional changes are expected to benefit the diagnosed individuals by preventing more severe disease or future complications. The benefit of labeling “ASD” well-functioning or unwilling individuals, as is often the case for children and adolescents, is questionable. The diagnosis may raise anxiety and stigmatization, or harm self-esteem during crucial periods of identity building. For instance, the quest for an autism diagnosis, less stigmatizing than a borderline personality disorder, represents an inadequate and misleading subjective identification of the individual with this later condition. The inclusion of subclinical individuals in a large and heterogeneous category minimally helps in decisions concerning support: intervention strategies are driven by specifier values as much as by common, but hypothetical,^5^ neurobiological substrates. Moreover, it leads to the overwhelming of specialized services and risks diverting attention and resources away from those who would benefit the most.

## Developmental Issues of Diagnosis

The boundaries of autism are fuzzy and unstable at both ends of life, from the early first signs to the outcome in adulthood.^9^ International recommendations favor the involvement of pediatricians in early detection during the regular and systematic follow-up of infants because of the expected benefits of early intervention. The reality of such a universal benefit has not been demonstrated. The relationship between what is detectable in the first year of life in prospective studies on “at-risk” infants and autism may be uncertain, in particular, when an additional neurogenetic condition is found. Screening instruments have been proposed, among which the modified checklist for autism in toddlers (M-CHAT) is the most widely used parental questionnaire. Its positive predictive value is good for neurodevelopmental disorders as a whole, but poorly specific for autism. Conversely, the M-CHAT has lower sensitivity than previously thought, as clinical impairment of intelligent and verbal autistic children may only become detectable later, due to growing social demands.

At school and adult age, the prioritization of ASD diagnosis and knowledge by health and teaching professionals, relative to that for other frequently comorbid conditions or those superficially resembling autism, overshadows the recognition and awareness of these conditions. At the other end of life, certain adults whose autism diagnosis was given in childhood no longer meet the diagnostic threshold, leading to discussions about optimal outcomes versus a potential erroneous former diagnosis. Autistic features may also fluctuate around and below the threshold value for ASD from 1 period of life to another. Autistic traits may be blurred and other clinical signs may come to the fore, modifying the picture and the needs of the individual. For instance, relationship difficulties can fade and become difficult to observe through developmental transformation and not, as predominantly considered, through the predominant action of camouflage: the notion of a “female profile” in autism has gained more ground in the general public than is actually supported by data.

On the other hand, specific learning or concentration difficulties can become predominant in terms of daily difficulties or as an anxio-depressive picture appears. For all these reasons, an evaluation of adaptive behavior should be prioritized in every diagnostic process for neurodevelopmental disorders, but without rushing to an ASD diagnosis. It may sometimes be prudent to consider “autism risk” or a “working diagnosis” and to regularly reassess the diagnosis. A categorical and definitive diagnosis is not obligatory to start the interventions, which are all the more motivated by the individual functioning than by the diagnosis as one moves away from a prototypical picture.

## Comorbidities and Complex Situations

The dimensional approach advocated by the DSM-5 aimed to avoid classifying people into excessively narrow, poorly reliable categories. Unfortunately, its current “spectrum” formulation creates another type of problem, that of differential diagnosis when the clinical presentation is barely distinguishable from that of the typical population or that of neurodevelopmental disorders. Many children who display syndromic presentations underpinned by various underlying mechanisms fulfill a sufficient number of criteria to be included in the spectrum.^10^ Numerous genetic, antenatal, or perinatal causes have also been implicated in complex neurodevelopmental disorders, including ASD clinical signs currently encompassed by the “spectrum” category. These conditions are markedly heterogeneous in terms of etiology, clinical presentation, outcome, and, above all, needs. For example, individuals with Fragile X or Cornelia De Lange (CDL) syndrome present with more severe autistic-like presentations than those with “Cri du chat” Syndrome. Social deficits tend to worsen after 15 years of age in CDL syndrome, whereas restricted and repetitive behavior improves over time in Fragile X syndrome. ASD diagnosed in premature babies is qualitatively different from that in full-term babies, but their autism diagnostic observation schedule score may not differ. Conversely, the same etiology may manifest in multiple, pleiotropic presentations for most “syndromic autism” presentations, whereas similar symptoms (e.g., low intelligence quotient) are shared by individuals harboring a large variety of genetic insults (e.g., deletions). Fragile X syndrome causes borderline intellectual functioning and/or ADHD and/or autism and/or oppositional disorder.

Unfortunately, only an ASD diagnosis unlocks access to preschool support in some countries and dedicated classes with a decent teacher/pupil ratio, of which the recruitment largely exceeds the scope of classic autism, whereas there is no equivalent for other conditions. In Quebec, for example, a large and autonomous network of specialized schools and assessment services is dedicated to ASD. The intensity of intervention classically associated with and unlocked by this diagnosis is without an equivalent for other conditions, despite questionable empirical support. Finally, an ASD diagnosis increases the level of financial support for families in most advanced countries. However, this hierarchical position does not necessarily reflect the hierarchy of needs among neurodevelopmental conditions. The relative prominence of various deficits should be taken into account at the time of diagnosis, for this determines the person's needs and shapes the optimal intervention.

Therefore, we argue that encompassing such complex neurodevelopmental conditions under a common ASD diagnosis may sometimes lead to inappropriate or uninformative decisions. In the absence of a description of the strengths and weaknesses of the individual, or for cases of clinical presentation far from the prototypical description of the category used to classify the patient, diagnosis-based interventions may neglect, or even contradict, an individual's actual sources of concern. Similarly, psychiatric comorbidity (e.g., depression or anxiety) may be more damaging to the quality of life for non-syndromic autistic presentations than the particularities explained by autism per se.

In complex situations that include multiple comorbidities, we recommend that clinicians do not prioritize deciding whether a particular individual is within or outside the autism spectrum, but rather refer to the diagnosis of “other specified or unspecified NDD,” while describing the neurodevelopmental and adaptive profile underlying the individual's strengths and weaknesses. Moreover, the use of a severity score for the developmental/behavioral areas under concern, and not the diagnosis would allow the family or adult individual to understand the uniqueness of the assessed person. Such information would allow the professional in charge to create an intervention plan and the public authorities to grant aid and support, to any areas of need rather than only on “social” and “repetitive” areas on which the diagnosis is built.

## Nine Principles to Guide Clinicians in Situations in Which the Autism Diagnosis is Debatable

- *Recognize autism.* Give more weight to a combination of qualitatively recognizable signs (e.g., odd language and prosody, rigid posture and gait, motor stereotypies, atypical visual exploratory behavior, and hand-leading) and trajectory (language regression) than to quantitative, abstractly defined deficits (e.g., social reciprocity impairment).
- *Combine standardized instruments with clinical expertise.* The clinical impression of expert clinicians should be prioritized when the interpretation of borderline scores raises uncertainties.
- *Do not confuse severity with prototypicality*. An evaluation of adaptative behavior is recommended, in addition to, but independently of, the diagnostic process. Autism can be prototypical or marginally representative of the category and orthogonal to functional impairment, which can run from moderate to severe.
- *Do not rely on the diagnosis for support*. The person's needs are more tightly linked to their functional impairment and adaptative behavior than to the diagnosis. The person's needs may differ more within the current ASD category than between ASD and other neurodevelopmental conditions.
- *“Autistic traits” are not autism*. If behavioral or cognitive features that evoke autism are barely visible and isolated, do not correspond to a deep and prolonged subjective feeling of difference, or are present amongst multiple and complex non-autistic symptoms, an ASD diagnosis has no added interventional value.
- *Consider the present and/or past visibility of the presentation*. Behavioral or emotional atypicalities are likely to have been noticed for a prolonged period at one moment or another during the individuals’ developmental trajectory. In adults, access to family testimonials is recommended. Specific attention should also be paid to qualitatively specific signs when looking for early signs and dysfunction.
- *A description is more relevant than a label in complex and atypical presentations.* A detailed neurodevelopmental and cognitive profile may be more informative than a diagnosis of ASD or the causative mutation or syndrome. If necessary, use the diagnosis of “other specified or unspecified neurodevelopmental disorder” complemented by a precise and individual description of functioning.
- *A delayed and/or provisional diagnosis associated with periodic reassessments may be the right choice.* Early identification of difficulties and atypicalities is recommended in all neurodevelopmental disorders, but without rushing to an ASD diagnosis and without delaying intervention.
- *Question the subjective and societal utility of the diagnosis*. It is worth weighing the pros and the cons of a diagnosis of ASD, especially for children and adolescents, whose proper consent is often difficult to obtain. When the diagnosis risks harm, it may be postponed. Always consider the best interest of the individual and his own choice.
